# A prediction nomogram for suboptimal debulking surgery in patients with serous ovarian carcinoma based on MRI T1 dual-echo imaging and diffusion-weighted imaging

**DOI:** 10.1186/s13244-022-01343-z

**Published:** 2022-12-27

**Authors:** Li Liu, Jie Wang, Yan Wu, Qiao Chen, Linyi Zhou, Hua Linghu, Yongmei Li

**Affiliations:** 1 Department of Radiology, The First Affiliated Hospital of Chongqing Medical University, No. 1 Youyi Road, Yuanjiagang, Yuzhong District, Chongqing, 400016 China; 2 Department of Radiology, The People’s Hospital of Yubei District of Chongqing City, No. 23 ZhongyangGongyuanBei Road, Yubei District, Chongqing, 401120 China; 3 Department of Nuclear Medicine, The First Affiliated Hospital of Chongqing Medical University, No. 1 Youyi Road, Yuanjiagang, Yuzhong District, Chongqing, 400016 China; 4 Nursing School of Chongqing Medical University, No.1 Medical College Road, Yuzhong District, Chongqing, 400016 China; 5 School of Public Health, Chongqing Medical University, No.1 Medical College Road, Yuzhong District, Chongqing, 400016 China; 6 Department of Radiology, Daping Hospital, Army Medical Center, Army Medical University, 10# Changjiangzhilu, Chongqing, 40024 China; 7 Department of Obstetrics and Gynecology, The First Affiliated Hospital of Chongqing Medical University, No. 1 Youyi Road, Yuanjiagang, Yuzhong District, Chongqing, 400016 China

**Keywords:** Prediction nomogram, Suboptimal debulking surgery, Serous ovarian carcinoma, MR-T1 dual-echo imaging, External validation

## Abstract

**Background:**

Serous ovarian carcinoma (SOC) has the highest morbidity and mortality among ovarian carcinoma. Accurate identification of the probability of suboptimal debulking surgery (SDS) is critical. This study aimed to develop a preoperative prediction nomogram of SDS for patients with SOC.

**Methods:**

A prediction model was established including 205 patients of SOC from institution A, and 45 patients from institution B were enrolled for external validation. Multivariate logistic regression was used to screen independent predictors and establish a nomogram to predict the occurrence of SDS.

**Results:**

Multivariate logistic regression demonstrated that the CA-125 level (odds ratio [OR] 8.260, 95% confidence interval [CI] 2.003–43.372), relationship between the sigmoid colon/rectum and ovarian mass (OR 28.701, 95% CI 4.561–286.070), diaphragmatic metastasis (OR 12.369, 95% CI 1.675–274.063), and FIGO stage (OR 32.990, 95% CI 6.623–274.509) were independent predictors for SDS. The area under the curve, concordance index, and 95% CI of the nomogram constructed from the above four factors were 0.951, 0.934, and 0.919–0.982, respectively. The model showed a good fit by the Hosmer–Lemeshow test (training set, *p* = 0.2475; internal validation set, *p* = 0.2355; external validation set, *p* = 0.2707). The external validation proved the reliability of the prediction nomogram. The calibration curve was close to the ideal diagonal line. The decision curve analysis demonstrated a significantly better net benefit. The clinical impact curve indicated good effectiveness in clinical application.

**Conclusion:**

A prediction nomogram for SDS in patients with SOC provides gynecologists with an accurate and effective tool for appropriate management.

## Key points

- The MRI-based nomogram could predict the SDS occurrence in patients with SOC.
- The relationship between the sigmoid colon/rectum and ovarian mass accounts is critical.
- A preoperative nomogram provides gynecologists with an accurate and effective tool.

## Introduction

Ovarian carcinoma (OC) has the highest mortality rate among patients with gynecological malignant tumors [1, 2], and serous ovarian carcinoma (SOC) has the highest morbidity and mortality rates [3, 4]. Most patients with SOC are often diagnosed at an advanced stage [5] as the mass is hiding in the deep pelvis, for whom primary debulking surgery (PDS) followed by platinum-based chemotherapy or neoadjuvant chemotherapy (NACT) followed by interval debulking surgery (IDS) has been the standard therapeutic strategy. Optimal debulking surgery (ODS, no residual disease) can prolong the progression-free survival and improve the prognosis of patients with SOC. However, 25–90% of patients cannot achieve ODS [6], and a significant proportion of patients who undergo suboptimal debulking surgery (SDS) have no significant improvement in survival [7]. The residual disease is a significant factor that affects the chemotherapy response rate and survival rate of patients with OC [8, 9]. Additionally, the clinical outcome of PDS followed by platinum-based chemotherapy is superior to NACT followed by IDS for patients with OC at the International Federation of Gynecology and Obstetrics (FIGO) stage IIIC or IV according to the American Society of Gynecological Oncology [10]. Therefore, identifying patients whose surgical outcomes might achieve SDS before initial treatment and reducing patients’ unnecessary tumor reduction surgery to choose PDS or NACT followed by IDS have always been the focus of academic research.

Many gynecologists have made various efforts to find a method or establish a model to predict SDS to guide therapeutic strategies. Previous studies have concentrated mainly on tumor markers, image methods, and laparoscopic exploration. Among them, the assessment of the ability of preoperative computed tomography (CT) and serum cancer antigen 125 (CA-125) by Suidan et al. [11] was the most representative model. However, it included three clinical and eight radiological criteria. The radiological criteria were complex and acquired a deeper understanding of image findings, which relied on the radiologist’s experience. Li et al. [12] established a radiomic-clinical nomogram based on magnetic resonance imaging (MRI) to predict residual disease for high-grade SOC. This nomogram solved the problem of radiologist experience, but it did not consider the contribution of abdominal metastases to surgical outcomes. Moreover, laparoscopic exploration enables gynecologists to assess residual lesions clearly, but it undoubtedly increases the economic and physical burden of patients with SOC.

To date, few studies have focused on the evaluation of SDS based on MRI T1 dual-echo imaging (DEI) combined diffusion-weighted imaging (DWI). In this study, we integrated MRI-T1-DEI, DWI, and several clinical factors to develop a prediction model and performed external validation to investigate whether it could improve the predictive accuracy of SDS in patients with SOC effectively, and if so, it will confer great clinical value.

## Materials and methods

### Patients

The study protocol was approved by the ethics review committees of institutions A and B, and the requirement of written informed consent was waived from all patients because of the retrospective nature of the study.

From January 2016 to December 2020, a total of 2565 patients with ovarian neoplasm in institution A were included initially. The inclusion criteria were patients who underwent MRI that ranged from the top of the diaphragm to the inferior pubic symphysis and performed PDS at institution A. Subsequently, patients enrolled were further screened according to the following exclusion criteria: (1) confirmed non-SOC pathologically, (2) received NACT or other anti-oncologic therapies before MRI examination and PDS, (3) had an interval of > 1 month between MRI/laboratory results and subsequent surgical pathological analysis, and (4) had incomplete clinical data. By searching the picture archiving and communication system (PACS) of institution B, we initially enrolled 497 patients with ovarian neoplasm from January 2019 to March 2022. The inclusion and exclusion criteria were the same as that in institution A. Subsequently, 45 patients from institution B were screened as the external validation set. The clinical data of all patients screened in the two institutions were collected, including age, menopausal status, laboratory results (CA-125, serum human epididymis protein 4 [HE-4], serum lactate dehydrogenase [LDH] level, and neutrophil-to-lymphocyte ratio [NLR]), American Society of Anesthesiologists Classification, FIGO stage (I/II & III/IV), and debulking results (ODS/SDS). ODS was deemed as R0, whereas SDS was deemed as R1 (a residual disease with a maximum diameter of ≤ 1 cm) or R2 (a residual disease with a maximum diameter of > 1 cm). The debulking results were assessed by a gynecologist with > 20 years of experience in gynecologic tumor debulking surgery according to surgical records or videos.

### MRI protocols

Abdominal and pelvic MRI examinations were conducted using the 3.0 Tesla whole-body MRI system in institution A (Signa HDxt, GE Medical Systems, Milwaukee, Wisconsin). All patients underwent MRI using a body phased-array coil that ranged from the top of the diaphragm to the inferior pubic symphysis, which was completed in two batches. Imaging sequences were as follows: axial T1-DEI (flip angle/time of repetition [TR], 80°/265 ms); slice thickness, 5 mm; gap, 1 mm; field of view, 40 cm; and NEX, 0.5. DWI was performed in the axial planes with a b value of 800 s/mm^2^ using spin-echo echo-planar imaging (SE-EPI; TR/time of echo [TE], 5500 ms/63.9 ms). In institution B, all patients also underwent abdominal and pelvic MRI examinations using the 1.5 Tesla MRI system (Signa HDxt, GE Medical Systems), and the imaging parameters were as follows: axial T1-DEI (flip angle/TR, 80°/200 ms); slice thickness, 5 mm; gap, 1 mm; field of view, 40 cm; and NEX, 0.75. DWI used SE-EPI (TR/TE, 4000 ms/74.8 ms), with a b value of 800 s/mm^2^.

### Image data collection

Two experienced radiologists (L.L. and Y.M.L with > 10 years of experience in abdominal imaging) analyzed the MRI features on the PACS workstation. Any disagreement was finally resolved through consultation. Our study only included conventional MRI sequences; as a result, the MRI scans (3.0 or 1.5 T) do not differ in the assessment of image variables in our model. Based on previous studies [11, 13–16], the following MRI features were carefully observed and recorded: (1) Ovarian mass features, namely, solid (the solid component accounted for more than two-thirds), complex cystic and solid (the solid component accounted for one- to two-thirds), and mainly cystic (the solid component accounted for less than one-third); (2) relationship between the sigmoid colon/rectum and ovarian mass or mass implanted in Douglas’ pouch on MR-T1-DEI (referred to as the relationship); we classified it into four grades: 0, clear (a hook edge sign existed between the sigmoid colon/rectum and ovarian mass or mass implanted in Douglas’ pouch), which means that the boundary between the two is clear; 1, close (the hook edge disappeared, but the shape of the sigmoid colon/rectum and mass can be vaguely distinguished); 2, bridge sign (the hook edge disappeared, and the two were limited adhered); and 3, fusion (the hook edge disappeared, and the two fused into a block); (3) metastases of distant organs in the abdomen; (4) bladder invasion; (5) diaphragmatic metastasis; (6) nodules or masses implanted on the omentum/peritoneum; (7) hydroureter; (8) retroperitoneal lymphadenectasis; and (9) amount of ascites (small, defined as ascites confined to the pelvic; medium-to-large, defined as ascites beyond the pelvis).

### Statistical analysis

All statistical analyses were conducted using R version 4.20 and SAS 9.4 software. Continuous variables with a normal distribution are presented as means ± standard deviations, whereas nonnormally distributed variables are presented as median (third quartile–first quartile). Student’s t tests and Wilcoxon’s tests were used to compare continuous variables. Person’s chi-squared and Fisher’s exact tests were used to compare categorical variables, which are presented as absolute numbers (%). Then, single-factor logistic regression was used to transform the continuous variables into categorical variables, and Youden’s index was used to determine the optimal cutoff point. Univariate analysis was used to screen each of the clinical and radiological variables. Subsequently, all variables with a significant difference were calculated via multivariate analysis to evaluate independent predictors.

Furthermore, logistic regression was used to construct a nomogram model to predict the occurrence of SDS. The receiver operating characteristic (ROC) curve, area under the ROC curve (AUC), concordance index (C-index), and calibration curve were used to evaluate the predictive accuracy and conformity of the model. The Hosmer–Lemeshow test was used to assess the goodness of fit of the model. The decision curve analysis (DCA) reflected the net benefit of the model for patients. *p* < 0.05 was considered a significant difference. Both discrimination and calibration were assessed by bootstrapping with 500 resamples. Finally, the clinical impact curve was used to predict risk stratification among 1000 people to predict the effectiveness of the model in clinical application.

## Results

### Flow diagram and general characteristics

**Fig. 1:**
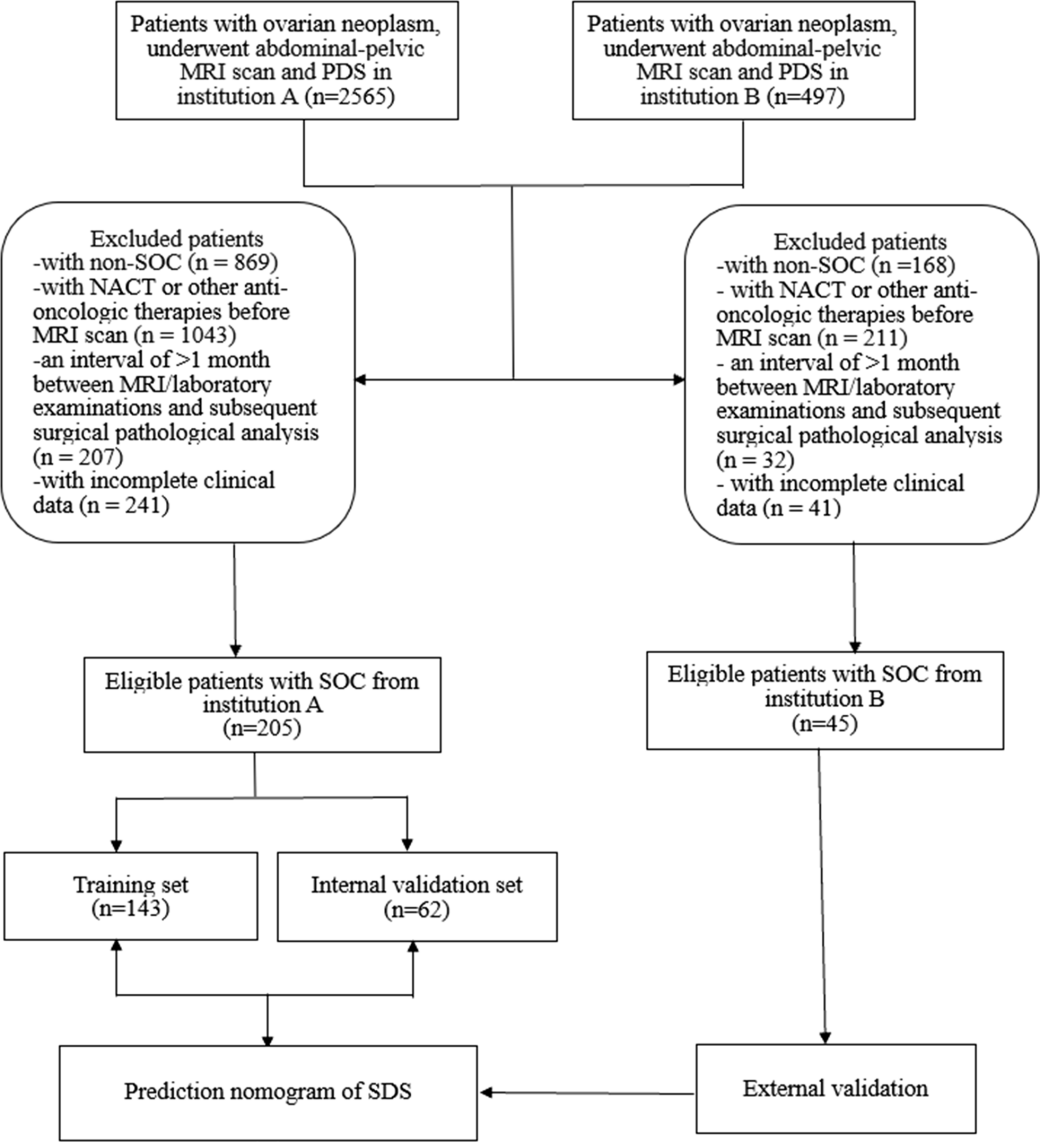
Flow diagram for this study

A total of 2565 patients with ovarian neoplasm who underwent abdominal and pelvic MRI and PDS in institution A were initially enrolled, and 497 patients with ovarian neoplasm from January 2019 to March 2022 in institution B according to the same inclusion criteria were initially enrolled. Ultimately, a total of 205 patients from institution A and 45 patients from institution B were included according to the selection criteria. Figure 1 presents the flow diagram for this study.

The patients from institution A were allocated into two sets: 143 patients in the training set and 62 in the internal validation set at a ratio of 7:3 using computer-generated random numbers, as shown in Tables 1 and 2. No significant differences were found in other variables in the training and internal validation sets (*p* > 0.05), except for distribution (*p* < 0.05). Single-factor logistic regression was used to transform partial continuous variables into categorical variables, and Youden’s index was used to determine the optimal cutoff point: age (cutoff = 45, C-index = 0.639), CA-125 (cutoff = 1484, C-index = 0.654), HE-4 (cutoff = 241, C-index = 0.558), LDH (cutoff = 227, C-index = 0.652), and NLR (cutoff = 3.56, C-index = 0.674).

**Table 1:** Clinical characteristics of patients with SOC from institution A

Characteristics	Training set, *n* = 143	Internal validation set, *n* = 62	*p* value
Debulking results			0.8045^b^
R0	48 (33.57)	19 (30.65)	
R1 + R2	95 (66.43)	43 (6935)	
Clinical characteristic			
Age	53.92 ± 8.76	53.84 ± 10.54	0.956^a^
CA-125	923.2 (317.5, 2795.8)	1466.0 (538.5, 2762.8)	0.4374^c^
HE-4	362.0 (156.0, 754.0)	422.5 (180.20, 803.8)	0.469^c^
LDH	218.0 (169.5, 346.0)	226.50 (171.5, 280.5)	0.8265^c^
NLR	3.33 (2.12, 4.77)	3.275 (2.243, 4.827)	0.9265^c^
Menopausal status			0.5322^b^
Premenopause	45 (31.47)	23 (37.10)	
Postmenopause	98 (68.53)	39 (62.90)	
FIGO stage			0.657^b^
I/II	24 (16.80)	12 (19.40)	
III/IV	119 (83.20)	50 (80.60)	
ASA			0.293^b^
I/II	98 (68.53)	47 (75.81)	
III/IV	45 (31.47)	15 (24.19)	

*SOC* Serous ovarian carcinoma, *R0* no residual disease, *R1* a residual disease with a maximum diameter of ≤ 1 cm; *R2* a residual disease with a maximum diameter of > 1 cm; *FIGO* International Federation of Gynecology and Obstetrics, *ASA* American Society of Anesthesiologists Classification

^a^Two independent samples Student’s t test

^b^Chi-squared test

^c^Wilcoxon’s test

**Table 2:** MRI characteristics of patients with SOC from institution A

Characteristics	Training set, *n* = 143	Internal validation set, *n* = 62	*p* value
Distribution			0.007
Unilateral	77 (53.85)	21 (33.87)	
Bilateral	66 (46.15)	41 (66.13)	
Mass feature			0.8876
Solid	79 (55.24)	34 (54.84)	
Complex cystic and solid	40 (27.97)	16 (25.81)	
Mainly cystic	24 (16.78)	12 (19.35)	
Relationship between the sigmoid colon/rectum and mass			0.9504
0 (clear, a hook edge existed)	27 (18.88)	11 (17.74)	
The hook edge disappeared			
1 (Close)	19 (13.29)	7 (11.29)	
2 (Bridge sign)	61 (42.66)	29 (46.77)	
3 (Fusion)	36 (25.17)	15 (24.19)	
Bladder invaded			0.4569
No	132 (92.31)	59 (95.16)	
Yes	11 (7.69)	3 (4.84)	
Metastases of distant organs			0.7651
No	131 (91.61)	56 (90.32)	
Yes	12 (8.39)	6 (9.68)	
Diaphragmatic metastasis			0.8327
No	99 (69.23)	42 (67.74)	
Yes	44 (30.77)	20 (32.26)	
Nodules or masses implanted on the omentum/ peritoneum			0.9487
No	34 (23.78)	15 (24.19)	
Yes	109 (76.22)	47 (75.81)	
Hydroureter			0.385
No	141 (98.6)	60 (96.77)	
Yes	2 (1.4)	2 (3.23)	
Retroperitoneal lymphadenectasis			0.2928
No	131 (91.61)	60 (96.77)	
Yes	12 (8.39)	2 (3.23)	
Amount of ascites			0.9733
No ascites or small	55 (38.46)	24 (38.71)	
Medium to large	88 (61.54)	38 (61.29)	

Chi-squared test

Table 3 provides the clinical and MRI characteristics of the patients with SOC from institution B.

**Table 3:** Clinical and MRI characteristics of patients with SOC from institution B

Characteristics	ODS group	SDS group
Clinical characteristics		
Age	52.95 ± 11.01	55.27 ± 5.42
Menopausal status		
Premenopause	4 (21.05)	4 (15.38)
Postmenopause	15 (78.95)	22 (84.62)
CA125	931.9 (391.90, 1384.22)	1000.50 (404.88, 2526.00)
HE4	260 (129.60, 608.00)	694.5 (449.00, 1097.00)
LDH	299.2 (200.60, 395.40)	424.45 (269.20, 545.40)
NLR	3.52 (2.04, 4.19)	4.49 (3.28, 6.77)
ASA		
I–II	19 (100)	19 (73.08)
III–IV	0	7 (26.92)
FIGO stage		
I–II	6 (31.58)	1 (3.85)
III–IV	13 (68.42)	25 (96.16)
MRI characteristics		
Distribution		
Unilateral	12 (63.16)	12 (46.15)
Bilateral	7 (36.84%)	14 (53.85)
Mass feature		
Solid	9 (47.37)	12 (11.54)
Complex cystic and solid	8 (42.11)	11 (42.31)
Mainly cystic	2 (10.53)	12 (46.15)
Relationship between the sigmoid colon/rectum and mass		
0 (clear, a hook edge existed)	7 (36.84)	0
The hook edge disappeared		
1 (Close)	4 (21.05)	5 (19.23)
2 (Bridge sign)	7 (36.84%)	12 (46.15)
3 (Fusion)	1 (5.26)	9 (34.62)
Bladder invaded	2 (10.53)	0
Metastases of distant organs	0	3 (11.54%)
Diaphragmatic metastasis	4 (21.05)	16 (61.54)
Nodules or masses implanted on the omentum/peritoneum	9 (47.37)	25 (96.15)
Hydroureter	0	0
Retroperitoneal lymphadenectasis	5 (26.32)	1 (3.85)
Amount of ascites		
No ascites or small	12 (63.16)	9 (34.62)
Medium to large	7 (36.84)	17 (65.38)

*SOC* Serous ovarian carcinoma, *ODS* optimal debulking surgery, *SDS* suboptimal debulking surgery

### Screening for independent predictors

**Fig. 2:**
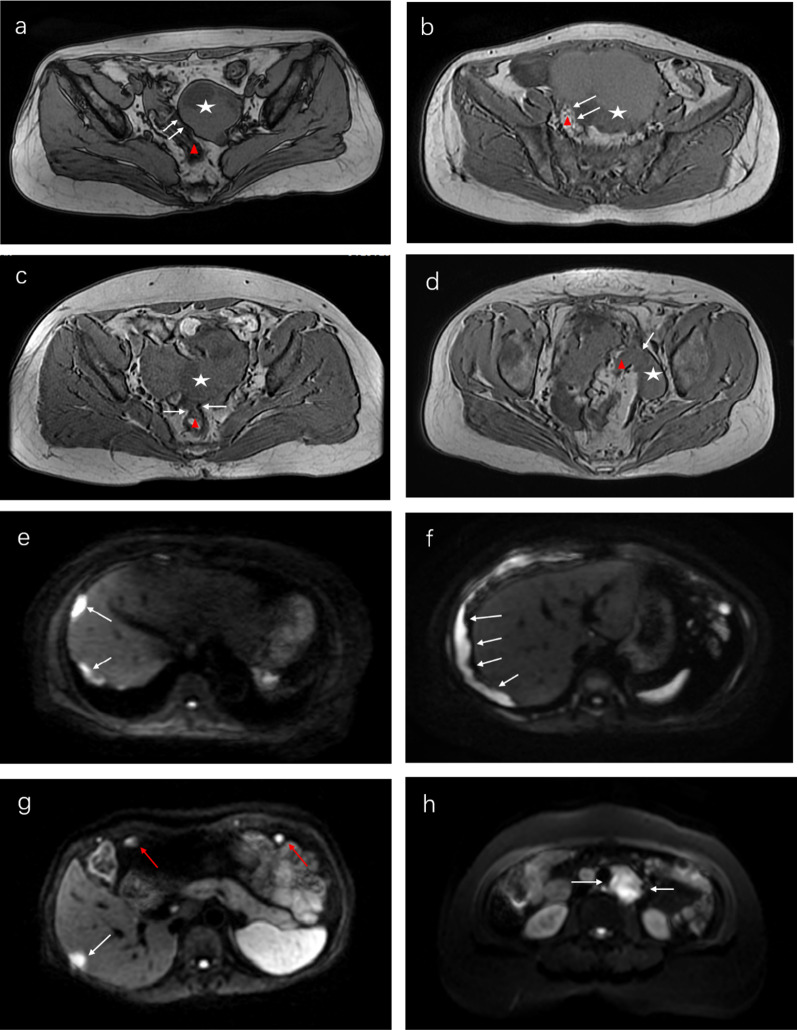
Image features of the MRI variables. **a**–**d** Four grades of relationship between the sigmoid colon/rectum and ovarian mass. **a** Axial MR T1 dual-echo (MR-T1-DE) image shows grade 0 (clear). A hook edge sign existed (white arrowheads) between the sigmoid colon (red triangle) and ovarian mass (white star). **b** Axial MR-T1-DE image shows grade 1 (close). The hook edge disappeared, but the shape of the sigmoid colon (red triangle) and ovarian mass (white star) can be vaguely distinguished (white arrowheads). **c** Axial MR-T1-DE image shows grade 2 (bridge sign). The hook edge disappeared, and the rectum (red triangle) and ovarian mass (white star) were limited adhered (white arrowheads). **d** Axial MR-T1-DE image shows grade 3 (fusion). The hook edge disappeared, and the sigmoid colon (red triangle) and ovarian mass (white star) fused into a block (white arrowheads). **e**, **f** Diaphragmatic metastasis. **e** Diffusion-weighted image shows hyperintense nodules implanted under the diaphragm (white arrowheads). **f** DW image shows extensive thickening of the diaphragm with hyperintensity (white arrowheads). **g** Metastases of liver and omentum. DW image shows hyperintense nodules of liver (white arrowhead) and omentum (red arrowheads). **h** Retroperitoneal lymphadenopathy. DW image shows significant hyperintensity of retroperitoneal enlarged lymph nodes (white arrowheads)

The univariate analysis demonstrated significant differences in the following 10 variables: CA-125, HE-4, LDH, NLR, FIGO stage, mass characteristics, amount of ascites, relationship between the sigmoid colon/rectum and mass, diaphragmatic metastasis, and nodules/masses implanted on the omentum/peritoneum (*p* < 0.05) (Fig. 2 and Table 4). Then, a multivariate logistic regression analysis based on the significant variables from the univariate analysis showed that four variables were independent predictors of SDS as follows: CA-125 level (*p* = 0.006, odds ratio [OR] 8.260, 95% confidence interval [CI] 2.003–43.372), relationship between the sigmoid colon/rectum and mass (*p* = 0.001, OR 28.701, 95% CI 4.561–286.070), diaphragmatic metastasis (*p* = 0.037, OR 12.369, 95% CI 1.675–274.063), FIGO stage (*p* = 0.0001, OR 32.990, 95% CI 6.623–274.509) (Table 5).

**Table 4:** Univariate analysis of clinical and MRI variables

Variables	N	*p* value
Age		
≤ 45 years	36	
> 45 years	169	0.2081
CA-125		
≤ 1484	118	
> 1484	87	< 0.001
HE-4		
≤ 241	78	
> 241	127	< 0.001
LDH		
≤ 227	107	
> 227	98	< 0.001
NLR		
≤ 3.56	115	
> 3.56	90	< 0.001
Menopausal status		
Premenopause	68	
Postmenopause	137	0.3806
FIGO stage		
I/II	36	
III/IV	169	< 0.001
ASA		
I/II	145	
III/IV	60	0.8418
Distribution		
Unilateral	98	
Bilateral	107	0.1395
Mass feature		
Mainly cystic	113	
Complex cystic and solid	56	0.0424
Solid	36	0.0040
Relationship between the sigmoid colon/rectum and mass		
0 (clear, a hook edge existed)	38	
The hook edge disappeared		
1 (Close)	26	< 0.001
2 (Bridge sign)	90	< 0.001
3 (Fusion)	51	< 0.001
Bladder invaded		
No	191	
Yes	14	0.1469
Metastases of distant organs		
No	187	
Yes	18	0.9647
Diaphragmatic metastasis		
No	141	
Yes	64	< 0.001
Nodules or masses implanted on the omentum/peritoneum		
No	49	
Yes	156	< 0.001
Hydroureter		
No	201	
Yes	4	0.9835
Retroperitoneal lymphadenectasis		
No	191	
Yes	14	0.1469
Amount of ascites		
No ascites or small	79	
Medium to large	126	< .0001

*FIGO* International Federation of Gynecology and Obstetrics, *ASA* American Society of Anesthesiologists Classification, *N* number

**Table 5:** Multivariate analysis of clinical and MRI variables

Variables	N	OR	95%CI	*p* value
CA-125				
≤ 1484	118	1		
> 1484	87	8.260	2.003–43.372	0.006
FIGO stage				
I/II	36	1		
III/IV	169	32.990	6.623–274.509	0.0001
Relationship between the sigmoid colon/rectum and mass				
0 (clear, a hook edge existed)	38	1		
The hook edge disappeared				
1 (Close)	26	0.624	0.092–3.876	0.611
2 (Bridge sign)	90	17.908	4.034–104.103	0.0004
3 (Fusion)	51	28.701	4.561–286.070	0.001
Diaphragmatic metastasis				
No	141	1		
Yes	64	12.369	1.675–274.063	0.037

*FIGO* International Federation of Gynecology and Obstetrics, *N* number, *OR* odds ratio

### Prediction nomogram establishment

**Fig. 3:**
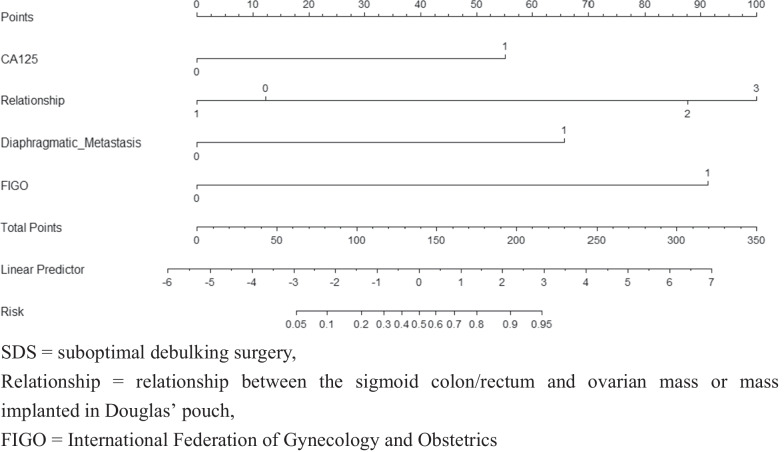
Nomogram for the prediction of SDS

The logistic regression model was constructed based on the above four variables (Table 5), and these four variables were integrated into the nomogram (C-index = 0.9509 [95% CI 0.919–0.982], bias C-index = 0.9356) (Fig. 3). For each patient, the higher the score, the higher the SDS risk. The Hosmer–Lemeshow test demonstrated the stable calibration of the prediction model (training set, *p* = 0.2475; internal validation set, *p* = 0.2355; external validation set, *p* = 0.2707). As can be seen from the nomogram, the relationship accounted for the highest weight, followed by the FIGO stage and diaphragmatic metastasis. Figure 2 shows the image features of the MRI independent predictors.

### Evaluation of the nomogram

**Fig. 4:**
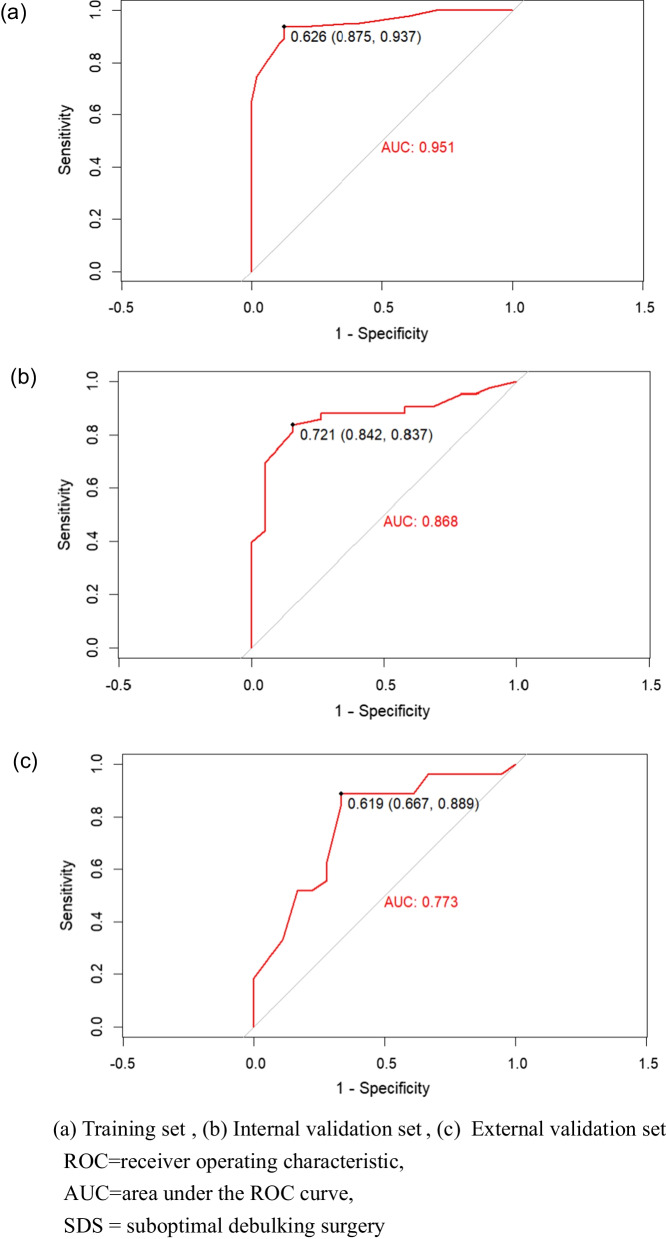
ROC curves of SDS

**Fig. 5:**
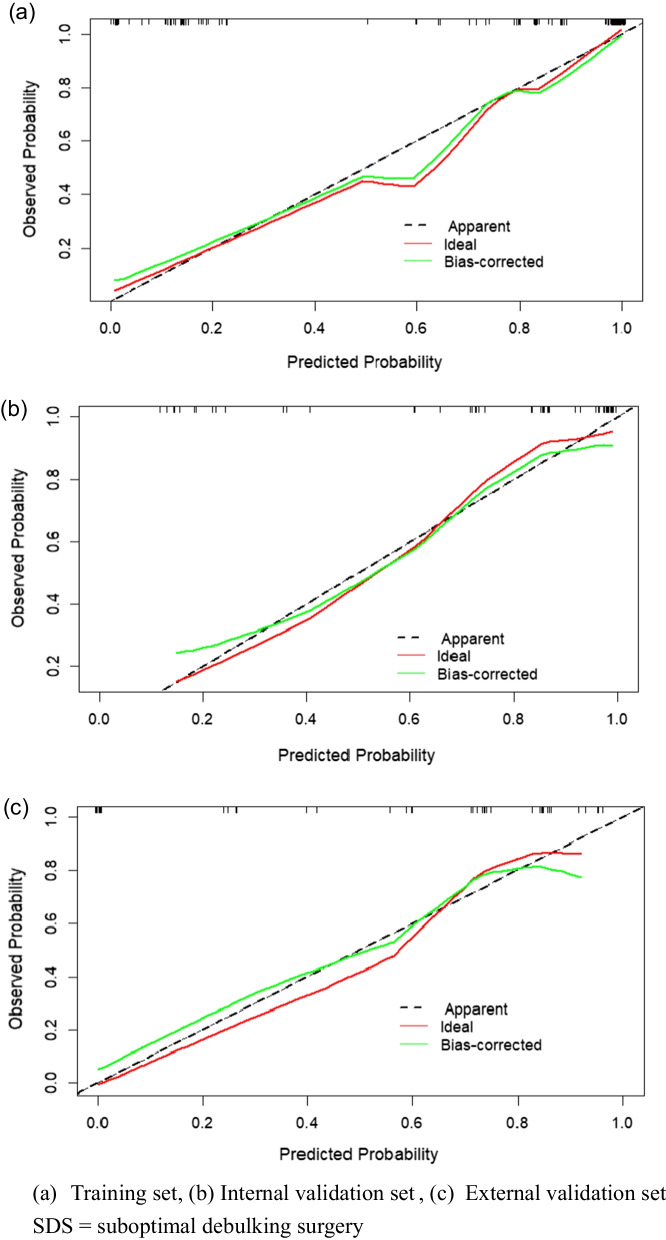
Calibration curve for predicting probability of SDS

**Fig. 6:**
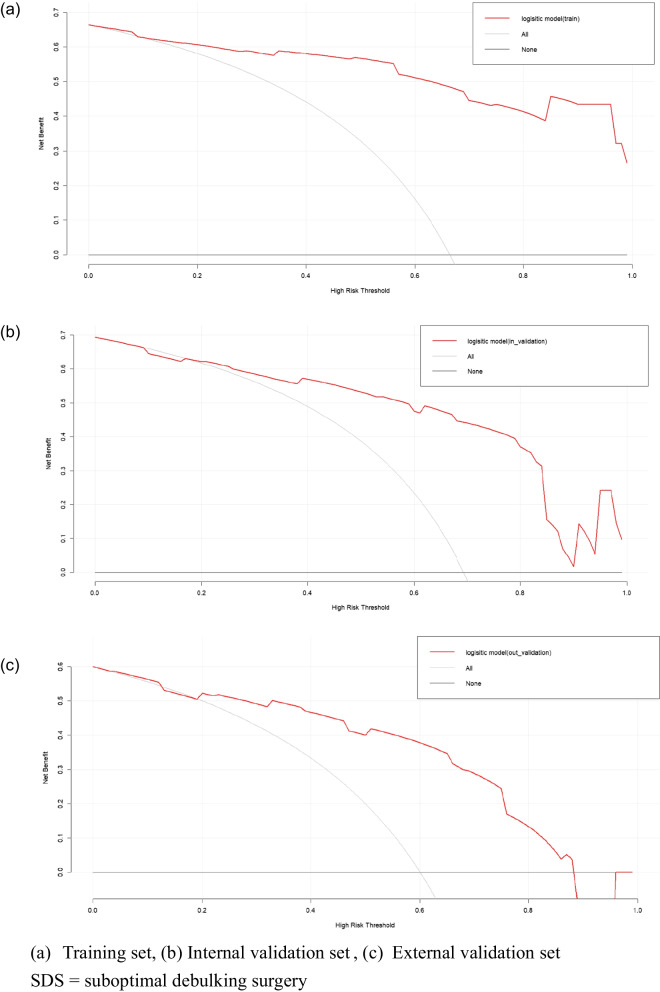
Decision curve analysis in prediction of SDS

**Fig. 7:**
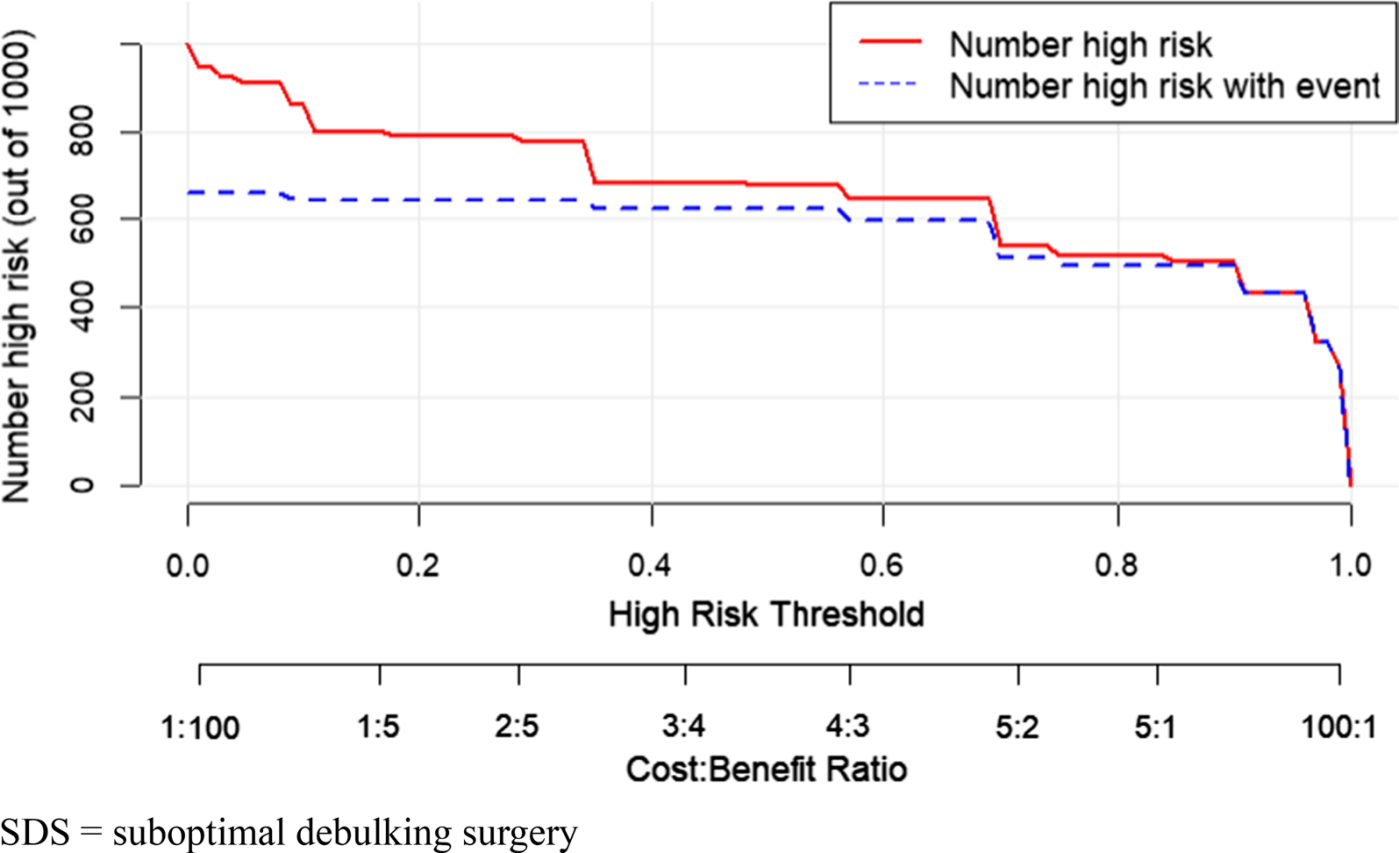
Clinical impact curve in prediction of SDS. *SDS* suboptimal debulking surgery

Figure 4 shows the ROC curves of the prediction model of the training, internal validation, and external validation sets, with AUCs of 0.951, 0.868, and 0.773, respectively, reflecting that the nomogram had good accuracy and consistency. Figure 5 provides the calibration curves of the three sets, which were all close to the ideal diagonal line. Furthermore, the DCAs showed a significantly better net benefit of the predictive model in the three sets (Fig. 6). The DCAs demonstrated when the risk of SDS is greater than 10%, intervention begins to achieve the clinical net benefit, and with the increase in risk, the clinical benefit of the intervention effect increases. As shown in the clinical impact curve (Fig. 7), the prediction model was used to predict risk stratification among 1000 people: the two curves were very close, indicating the good effects of the prediction model in clinical application.

## Discussion

Considering that different pathological types of OC have varied aggressiveness, to our knowledge, this study is the first to develop a nomogram for predicting the occurrence of SDS only in patients with SOC using several simple clinical and imaging variables based on MR-T1-DEI and DWI, whereas many studies about predicting SDS have selected cases of peritoneal carcinoma, fallopian tube cancer, advanced ovarian cancer, or epithelial ovarian cancer based on clinical and imaging factors in the past few decades. However, to our knowledge, these studies have focused on the metastasis site rather than on the relationship between metastasis and the site of spread, such as adhesion or fusion of adjacent organ. Our study revealed that the CA-125 level, the relationship between the sigmoid colon/rectum and ovarian mass or mass implanted in Douglas’ pouch, diaphragmatic metastasis, and FIGO stage were independent predictors of SDS in patients with SOC.

CA-125 is an important evaluation index in SOC. In models predicting SDS, most studies have included CA-125 as one of the variables. The cutoff value of CA-125 was 500 U/mL by Suidan [11], 800 U/mL by Yu Gu [14], and 420 U/mL by Maliheh [17]. However, the cutoff value of the present study was 1484 U/mL, which is significantly higher than those of the aforementioned studies. The possible reason is that all cases included in the present study were SOC, and the CA-125 levels of these cases were higher than those of other kinds of OC.

Our study also indicated that the relationship between the sigmoid colon/rectum and ovarian mass or mass implanted in Douglas’ pouch was an independent predictor of SDS, which was also included in the predictive model. Thus, this is the first evaluation of the relationship based on MR-TI-DEI, and we scaled it from 0 to 3 (Fig. 2). As shown in the nomogram (Fig. 3), the relationship accounted for the highest weight. The higher the grade of the relationship, the higher the points in the nomogram, and the higher the probability of SDS. These results are not difficult to understand. The metastasis site of OC is usually located in Douglas’ pouch, which often leads to adhesion or even fusion between the metastasis and the site of spread, increasing the difficulty of complete surgical tumor resection. If the tumor cannot be resected completely, rectal resection with enterostomy must be performed to achieve R0, but the quality of life of the patients must be significantly reduced. Since this is unacceptable for most patients, accurate assessment of the relationship before surgery is very important for debulking outcomes. As a result, we believe that it should be considered as an important variable affecting the surgical outcome, although invasion (fusion) of the tumor with sigmoid colon/rectum is already covered in the FIGO IIb stage. As in other research [16, 18, 19], diaphragmatic nodules and retroperitoneal lymphadenopathy were used as predictors of the SDS model. And our results confirm this view (Fig. 3).

As another independent predictor of SDS, diaphragmatic metastasis has been recognized by some researchers and included in prediction models [16, 18, 19]. Although the diaphragm has diaphragmatic muscles, it is still weak and adjacent to the thoracic cavity. Thus, debulking of diaphragmatic metastasis is dangerous, which increases the difficulty of PDS.

Moreover, the FIGO stage was an independent predictor of SDS and was included in the predictive model. Most patients with SOC are often diagnosed at an advanced stage as the mass hides in the deep pelvis. In our study, 7 cases of FIGO stage I, 29 cases of FIGO stage II, 134 cases of FIGO stage III, and 35 cases of FIGO stage IV were included. In our preliminary experiment, we tried to include FIGO according to the standard four stages, but there was no statistical significance by univariate analysis (*p* values were 0.988, 0.975, and 0.967, respectively). Considering that the early stage includes FIGO stages I and II and the advanced stage includes III and IV, we tried to dismember the standard stage into I/II & III/IV, namely early and advanced stages. By uni- and multivariate analyses, patients with preoperative FIGO stage I/II were more likely to achieve ODS than patients with preoperative FIGO stage III/IV (*p* < 0.05). This result reflects the importance of accurate preoperative FIGO staging for surgical outcomes.

In the selection of variables, we also included some other variables closely related to debulking surgery, such as nodules or masses of the omentum/peritoneum, metastases of distant organs in the abdomen, retroperitoneal lymphadenectasis (Fig. 2), bladder invasion, and hydroureter. However, according to the statistical results, they were not considered independent predictors. This may be because of the small number of related cases included. According to the statistical results in this study, HE-4, LDH, and NLR were not included as independent predictors, even though the latter two factors reflect systemic inflammation [20]. Therefore, this result still needs to be verified.

We relied primarily on MR-T1-DEI and DWI in evaluating some variables (location and neighboring relationship of implant metastasis). One of the innovations of this study is the evaluation of the relationship by taking advantage of MR-T1-DEI and grading them into four. The advantages of DWI in assessing intraperitoneal implantation metastasis in OC have been reported in some studies [15, 16, 21]. They demonstrated that DWI is superior to CT in the evaluation of OC metastatic lesions in the abdomen. On DWI (Fig. 2), intestinal contents and ascites were suppressed, and the intraperitoneal implantation metastatic lesions were obvious and easily observed, which not only makes evaluation by radiologists easier but also makes it more suitable for clinicians who are not good at radiology.

In this study, we assessed the preoperative objective, simple, and easily identifiable predictors of SDS and developed a risk prediction model. The AUCs of our model (training set = 0.951, internal validation set = 0.868, external validation set = 0.773) were higher than those of other models [12, 14, 18] (Fig. 4). Our internal and external validation confirmed the good accuracy and conformity of the model, alongside its net benefit. The nomogram is visual and personalized, which provides clinicians with a simple and intuitive tool for practical prediction.

However, this study has several limitations. First, there were four cases of ureteral invasion and 12 cases of bladder invasion that had not been a concern in previous studies; unfortunately, the sample size of these cases in our study was too small to be significant. We will continue to pay attention to these interesting findings in subsequent studies. Second, since this study is the first to use the relationship between the mass and rectum as a variable to explore its impact on surgical outcomes, we attempted to group the relationship more finely. In future studies, we will expand the sample size, further study the relationship between the mass and rectum, and explore better classification methods. Third, a degree of internal bias may be inevitable because of the retrospective nature. Although the ODS rate for advanced OC is between 35 and 92% [18, 22], we assume that newer imaging equipment and improved surgical techniques over time may allow for more sensible ODS rates. Therefore, it is necessary to continue this research and conduct some prospective studies in the future.

## Conclusions

In this study, we found that the CA-125 level, the relationship between the sigmoid colon/rectum and ovarian mass or mass implanted in Douglas’ pouch, diaphragmatic metastasis, and FIGO stage were independent predictors of SDS in patients with SOC. Based on these predictors, we created a preoperative prediction nomogram for SDS, and our external validation confirmed that this model was good. For each patient, higher total points reflected a greater risk of SDS. The visual and personalized model of preoperative predictors provides gynecologists with a simple and intuitive tool for preoperative evaluation of SDS, which may be of significance in the selection of the best treatment strategy and avoidance of unnecessary exploration surgeries.

## Data Availability

The datasets used and/or analyzed during the current study are available from the corresponding author upon reasonable request.

## Abbreviations

AUC: The area under the receiver operating characteristic curve
CA-125: Cancer antigen 125
C-index: Concordance index
CT: Computed tomography
DCA: Decision curve analysis
DWI: Diffusion-weighted imaging
FIGO: International Federation of Gynecology and Obstetrics
HE-4: Human epididymis protein 4
IDS: Interval debulking surgery
LDH: Lactate dehydrogenase
MRI: Magnetic resonance imaging
NACT: Neoadjuvant chemotherapy
NLR: Neutrophil-to-lymphocyte ratio
OC: Ovarian carcinoma
ODS: Optimal debulking surgery
PDS: Primary debulking surgery
ROC: Receiver operating characteristic
SDS: Suboptimal debulking surgery
SOC: Serous ovarian carcinoma
T1-DEI: T1 dual-echo imaging
